# The role of IL-1B in breast cancer bone metastasis

**DOI:** 10.1530/ERC-17-0309

**Published:** 2018-05-14

**Authors:** Claudia Tulotta, Penelope Ottewell

**Affiliations:** Department of Oncology and MetabolismMellanby Centre for Bone Research, University of Sheffield, Medical School, Sheffield, UK

**Keywords:** IL-1B, breast cancer, bone metastasis

## Abstract

Approximately 75% of patients with late-stage breast cancer will develop bone metastasis. This condition is currently considered incurable and patients’ life expectancy is limited to 2–3 years following diagnosis of bone involvement. Interleukin (IL)-1B is a pro-inflammatory cytokine whose expression in primary tumours has been identified as a potential biomarker for predicting breast cancer patients at increased risk for developing bone metastasis. In this review, we discuss how IL-1B from both the tumour cells and the tumour microenvironment influence growth of primary breast tumours, dissemination into the bone metastatic niche and proliferation into overt metastases. Recent evidence indicates that targeting IL-1B signalling may provide promising new treatments that can hold tumour cells in a dormant state within bone thus preventing formation of overt bone metastases.

## Introduction

Breast cancer bone metastasis is an incurable disease. Breast cancer is the most common cause of cancer death in females in Europe and worldwide. Although the mortality rate decreased by 35% since the early 1970s, it is estimated that one in eight females will develop breast cancer during their lifetime and the incidence rate is expected to increase by 2% in the United Kingdom by 2035 (Cancer Research UK, Cancer Statistics, July 2017).

Bone is the most common metastatic site for breast cancer, and bone metastases are associated with poor prognosis and significantly reduced life expectancy to just 2–3 years following diagnosis. Patients with bone metastasis experience increased morbidity due to severe pain, reduced mobility, hypercalcemia, osteolysis and bone fractures, as well as spinal cord compression and bone marrow aplasia. Bone pain is generally due to mechanical pressure exerted by a tumour cell mass or due to the release of inflammatory cytokines by either the tumour cells themselves or by the surrounding bone microenvironment, leading to altered bone homeostasis (Macedo* et al*. 2017).

The formation of breast cancer metastases in bone generally occurs in the axial skeleton, possibly in relation to high bone marrow content, where haematopoiesis actively takes place. The microenvironment is fundamental in attracting tumour cells to bone as well as promoting tumour progression. The composition of the tumour microenvironment changes during tumour development and is different at different sites (Hanahan & Weinberg 2011). This variation differently affects cancer progression as well as the efficacy of therapeutic regimes. A plethora of signalling molecules is involved in the intricate network of signalling pathways that orchestrate the communication between cancer cells and surrounding stroma. Interleukin-1B (IL-1B) is a pro-inflammatory cytokine whose expression in primary tumours has been identified as a potential biomarker for predicting breast cancer patients at increased risk for developing bone metastasis (Nutter* et al*. 2014).

Here, we provide a detailed overview of the molecular mechanisms underlying primary breast cancer development and formation of metastasis in bone, reporting recent evidence that suggest a pivotal role of IL-1B in these processes. Finally, we summarise current therapies that target the IL-1 signalling pathway that have the potential to be repurposed for the treatment of metastatic breast cancer.

## Breast cancer bone metastasis and the bone microenvironment

The bone is a preferred metastatic site for breast cancer cells. When tumour cells home to the bone, they utilise molecular mechanisms that control the interaction between different cell types including osteoblasts, osteoclasts and haematopoietic stem cells, altering bone homeostasis to promote tumour cell survival, dormancy and/or proliferation in this environment (Mishra* et al*. 2011). In bone, the bone marrow is the main site of haematopoiesis. Because haematopoetic stem cell (HSC) self-renewal and haematopoietic progenitor proliferation, commitment and differentiation occur in the same microenvironment, it is suggested that there must be different specific niches composed by different stromal cells that can regulate each step from HSC renewal to complete differentiation (Cordeiro-Spinetti* et al*. 2015). The bone marrow cavity can be divided into four niches: endosteal, subendosteal, central and perisinusoidal (Lord* et al*. 1975, Lambertsen & Weiss 1984). HSC and multipotent progenitors are found in the endosteal and subendosteal regions, whereas committed progenitors are located in the central region and the differentiated cells in the perisinusoidal niche (Cordeiro-Spinetti *et al*. 2015). Tumour cells home to specific regions in bone. It has been suggested that the bone microenvironment is a dynamic niche and that the formation of metastatic lesions resembles a constant osteogenesis process, allowing early-stage bone colonization. During this process, adherens junctions form between metastatic tumour cells, expressing E-cadherin, and cells in the bone niche, specifically osteogenic cells, expressing N-cadherin (Wang* et al*. 2015). It is hypothesised that tumour cells either remain dormant or are stimulated to proliferate into overt metastases dependent on which niches they interact with: Interactions between CXCR4, found on metastatic tumour cells, and CXCL12, expressed by osteoblasts, mesenchymal stem cells (MSCs) and adventitial reticular cells (Sugiyama* et al*. 2006) are a key component in homing and adhesion of tumour cells to the bone metastatic niche. In the endosteal niche, tumour cells are thought to be held in a quiescent state through CXCR4/CXCL12 interactions using similar mechanisms to those used by osteoblasts to maintain quiescence of HSCs (Wang* et al*. 2014). Tumour cells hijack mechanisms of HSCs mobilization and proliferation and form overt metastasis (Ottewell 2016). In addition, the role of CXCL12 in the primary tumour in the establishment of organ-specific metastasis has been described: Cancer-associated fibroblasts in the primary tumour mimic the bone marrow microenvironment by producing CXCL12, which has been found to enrich breast cancer Src-hyperactive clones, which survive in the bone marrow (Zhang* et al*. 2013). Src is responsible for mediating breast cancer cell survival in the bone marrow, by controlling tumour cell response to both pro-survival signals in the bone microenvironment like CXCL12 and by conferring resistance to pro-apoptotic signals such as TRAIL (Zhang* et al*. 2009).

**Figure 1:**
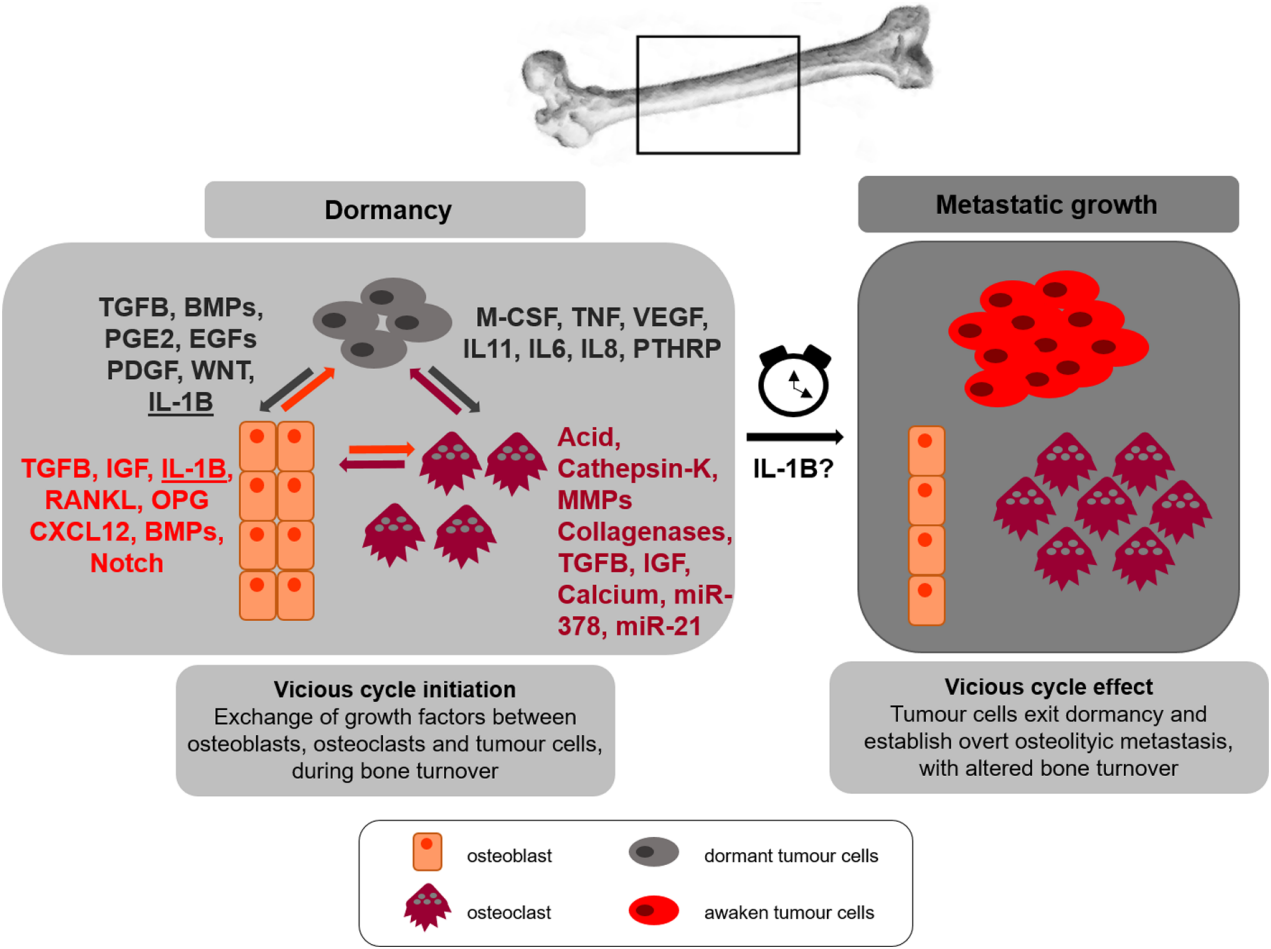
Vicious cycle and dormancy. Bone is a favourite site for metastatic breast cancer. Tumour cells arriving in bone undergo dormancy. Osteoclasts and osteoblasts secrete factors to maintain bone homeostasis (factors released by osteoblasts and osteoclasts are indicated in orange and purple, respectively). Bone remodelling cytokines stimulate tumour cells that, after homing in bone, have entered a dormant state. Simultaneously, tumour cells produce factors (in grey) that stimulate further osteoblasts and osteoclasts activity, resulting in awakening cancer cells with the formation of overt bone metastases and altered bone turnover. This vicious cycle leads to enhanced tumour cell proliferation and aggressiveness, increased osteoclast number and activity and decreased osteoblast number and function, resulting in osteolytic bone metastases. We hypothesise that IL-1B is a putative factor exchanged between tumour cells, osteoblasts and osteoclasts, and a trigger for tumour cells awakening from dormancy and formation of bone metastasis.

Breast cancer metastasis primarily manifests as a bone lytic disease; however, osteolytic, osteoblastic and mixed lesions occur. In the case of osteolytic metastases, tumour cells stimulate osteoclast activity and/or inhibit osteoblast differentiation, leading to increased bone resorption and/or decreased bone formation, respectively, finally resulting in the development of bone disease and higher risk of fractures. A vicious cycle between breast cancer cells and bone occurs and sustains the growth of tumour metastases. Growth factors, produced by osteoblasts, bind the bone matrix. Upon osteoclast-mediated resorption, growth factors are released into the local environment where they stimulate tumour cell growth. In turn, cancer cells themselves alter the function of both osteoblasts and osteoclasts, affecting bone turnover (Ottewell 2016) (Fig. 1). Molecules released by osteoblasts, osteoclasts and tumour cells during bone turnover have been extensively reviewed in (Weilbaecher* et al*. 2011, Le Pape* et al*. 2016, Ottewell 2016) and shown in Fig. 1. IL-1B plays a role in bone homeostasis. Inhibition of IL-1B prevents osteoclastogenesis and osteoblastogenesis, thus impairing bone resorption and bone formation, respectively (Nakamura & Jimi 2006, Ruscitti* et al*. 2015). IL-1B was shown to be produced by peripheral blood mononuclear cells (PBMCs) in premenopausal women who underwent ovariectomy and experienced a decrease in bone mineral density. Oestrogen administration restored IL-1B levels in these patients, thus suggesting that IL-1B has a major role in bone resorption upon oestrogen withdrawal (Pacifici* et al*. 1991). In osteoblastic metastases, tumour cells support osteoblastogenesis and osteoblast stimulation. In particular, transforming growth factor β (TGFB), bone morphogenetic proteins (BMPs) and WNT proteins are known to activate transcription factors, like RUNX2, that control osteoblast function. It is thought that metastatic tumour cells release such factors that drive osteoblast formation (Weilbaecher *et al*. 2011). Once activated, osteoblasts produce factors such as interleukin-6 (IL6), monocyte chemotactic protein-1 (MCP-1), vascular endothelial growth factor (VEGF) and macrophage inflammatory protein-2 (MIP-2) that sustain cancer growth and interaction with other cells in the bone microenvironment. Production of CXCL12, BMPs and Notch by osteoblasts sustains the recruitment of tumour cells in a similar manner to the physiological HSCs recruitment (Weilbaecher *et al*. 2011). In osteolytic metastases, TGFB and receptor activator of nuclear factor kappa-B ligand (RANKL) play a major role in driving tumour-bone vicious cycle, whereas BMP, epidermal growth factor (EGF) and platelet-derived growth factor (PDGF) signalling have been found to support this cycle in osteoblastic metastases (Ottewell 2016).

The formation of osteolytic lesions has been associated with extracellular matrix-modifying enzymes, lysyl oxidases (LOXs). In particular, LOXs are induced in hypoxic conditions in the primary tumour. Released in the circulation, LOXs travel to the bone where they impair osteoblast/osteoclast activity. LOXs cause increased bone resorption, by inhibiting osteoblast activity and increasing osteoclastogenesis. In this LOX-regulated environment, where pre-metastatic osteolytic lesions are formed, bone colonisation by breast cancer cells takes place (Cox* et al*. 2015, Sousa & Maatta 2016).

The activity of osteoblasts on bone is directly linked with the maintenance of breast cancer cell dormancy in this site. We recently showed that ovariectomy-induced increase in osteoclast activity and bone turnover stimulates metastatic breast cancer cells to exit from their dormant state and start proliferating. When osteoclast activity was reduced in this ovariectomy model using the RANKL/RANK inhibitor, osteoprotegerin (OPG), breast cancer cells disseminated into bone were unable to proliferate into overt metastases and remained in a dormant state. (Ottewell* et al*. 2015). A link between IL-1B and OPG has been recently found in breast cancer primary tumour development and metastasis and will be discussed later in this review.

Amongst other cell types present in the bone, osteocytes are considered to play pivotal functions in maintaining the balance between osteoclast and osteoblast activity during bone remodelling, by creating a multi-functional syncytium through the bone. Osteocytes are terminally differentiated cells derived from the osteoblast lineage. Osteoblasts are surrounded by their released extracellular matrix that forms the osteoid, constituted mainly by collagen I. Mature osteoblasts that localise in the osteoid are pre-osteocytes. During cell maturation, the osteoid mineralise and cells that are embedded in it are referred as osteocytes (Prideaux* et al*. 2016). In cancer biology, osteocytes have recently been described as playing anti-cancer functions. Osteocytes use connexin 43 (Cx43) hemichannels in an intrinsic self-defense mechanism against breast cancer cells colonizing the bone. Cx43 is used by osteocytes to allow intra- and extra-cellular passage of small molecules. Upon bisphosphonate treatment, Cx43 hemichannel opening results in anchorage-independent growth, migration and invasion inhibition of human breast cancer cells. Cx43-osteocyte-specific knock-out mice and osteocyte-specific transgenic mice with impaired Cx43 gap junctions and hemichannels displayed enhanced tumour growth, limiting the anti-tumour effect of bisphosphonate (ZOL) treatment. Release of ATP from Cx43 hemichannels in osteocytes was found to mediate anti-cancer functions (Zhou* et al*. 2016). Despite this evidence suggesting an anti-tumour function exerted by osteocytes through Cx43, *in vitro* studies have shown that osteocytes might also play pro-tumour functions, when different breast and prostate cancer cell lines are used. Indeed, breast cancer cell lines treated with conditioned medium from osteocytes displayed either increased or decreased invasion and chemotaxis (Cui* et al*. 2016). In addition, divergent roles of ATP and adenosine released by osteocytes on breast cancer cells support the dual role of this bone cell type on breast cancer bone metastases (Zhou* et al*. 2015).

ATP has been identified in the conditioned media of alendronate-treated osteocytes as a factor responsible for breast cancer cell migration inhibition. ATP exerts anti-cancer functions by activating purinergic P2X7 receptor on breast cancer cells. Use of ATP antagonist (oxidised ATP) or P2X7 knockdown results in reduced cancer inhibitory functions associated with ATP, whereas the use of BzATP agonist results in breast cancer migration inhibition *in vitro*. In addition, different ATP dosages correlate with pro- or anti-tumour function. In particular, low dosage of ATP inhibits breast cancer cell migration, whereas high dosage enhances breast cancer cell migration. In the same study, Zhou and colleagues showed that ATP significantly delays breast cancer primary tumour growth in a xenograft model. Finally, breast cancer growth in bone was also reduced *in vivo* upon treatment with a non-hydrolysable ATP (ATPγS). Interestingly, upon binding to P2X7R, ATP mediates NLRP3 inflammasome-dependent IL-1B secretion (Karmakar* et al*. 2016). Considering that IL-1B is a prognostic marker for breast cancer disease progression, it is puzzling to define a pro- or anti-tumour function for the energy-carrying molecule.

Alongside bone, the adipose tissue also presents support to breast cancer metastasis in this secondary site. During aging, adipose tissue increases in bone and, simultaneously, enhanced breast cancer metastasis can be observed. Breast cancer cells establish contacts with adipocytes when in co-culture with cancellous bone fragments of human origin and increase their migratory properties. The analysis of conditioned medium derived from human bone tissue identified high levels of IL-1B (Templeton* et al*. 2015).

More recent advances in the understanding of tumour–microenvironment interactions have elucidated the crucial role of the immune system in either supporting or inhibiting cancer progression. Tumour-associated macrophages (TAMs) have been found to infiltrate primary breast cancer. Previously classified in M1 (classically activated macrophages with anti-cancer functions) and M2 (alternatively activated macrophages with pro-tumour functions), macrophages are now grouped into four categories: M1 (IFNy/LPS), involved in inflammation, microbial killing and anti-tumour functions; M2a (IL4/IL13) involved in allergic reactions, intracellular parasite killing and matrix remodelling; M2b (immunocomplexes) associated with immunoregulation and suppression of inflammation and M2c (IL10) responsible for Treg stimulation, immunosuppression and matrix remodelling. TAMs generally have an M2 phenotype. In breast cancer, high density of TAMs associates with poor prognosis. TAMs support tumour angiogenesis, invasion by remodelling of the tumour extracellular matrix, mechanisms of immune evasion, infiltration of immunosuppressive leukocytes and activation of breast cancer stem cells (as reviewed by Choi* et al*. 2018). Moreover, in the context of tumour relapse after chemotherapy, MRC1^hi^, CXCR4^hi^, Tie2^hi^ VEGFA^+^ macrophages have been identified as tumour-supporting M2 macrophages localising in perivascular areas where CXCL12 is expressed. These are perivascular macrophages that stimulate tumour relapse after chemotherapy (Hughes* et al*. 2015). Tumour-infiltrating macrophages can be derived from circulating monocytes (formed in the bone marrow) or tissue-resident macrophages (embryonal origin). The role of bone marrow-resident macrophages (OsteoMacs) is currently under investigation. So far, indirect evidence allows to speculate that metalloprotease (MMP9) and macrophage colony-stimulating factor (M-CSF) levels in breast cancer cells (MDA-MB-231) correlate with TAM infiltration and OsteoMac polarization (Sousa & Maatta 2016). Monocytes and macrophages can differentiate into osteoclasts by the combination of RANKL and M-CSF. Additional stimulation with cytokines involved in M1/M2 polarisation has been found to either promote or impair macrophage differentiation into osteoclasts (Jeganathan* et al*. 2014). Therefore, understanding the balance between macrophage and osteoclast polarisation in bone could provide new insights in breast cancer bone metastases.

Interestingly, breast cancer cells have been found to modulate the haematopoietic stem cell niche by modulating the CXCL12–CXCR4 signalling axis. In particular, CXCL12 levels in bone marrow-derived mesenchymal stromal cells were found to be lower upon treatment with medium derived from breast cancer cells or in co-culture conditions. CXCL12 downregulation affected HSPCs migration towards MSC/cancer cells co-culture or conditioned medium (Wobus* et al*. 2015). Furthermore, the cross-talk between MSCs and cancer cells relates to CDH1 (E-cadherin) and IL-1B levels. High CDH1 and low IL-1B in cancer cells induce MSC organization into a niche like formation, dependent on direct cell–cell contact, *in vitro* (Al-toub* et al*. 2015).

It has been hypothesised that metastatic cancer cells compete with HSCs to colonise their niche in bone. Evidence from prostate cancer shows that CXCL12-CXCR4 signalling is involved in the niche colonisation by tumour cells and competition with HSCs. Once in bone, dormant tumour cells are exposed to a microenvironment that generally maintains HSCs in a quiescent state. This microenvironment is hypoxic and populated by stromal cells that produce anti-apoptotic and pro-survival signals (Ren* et al*. 2015). Therefore, tumour–HSC interaction mainly contributes to tumour dormancy.

## IL-1B signalling and breast cancer

### IL-1B signalling

**Figure 2:**
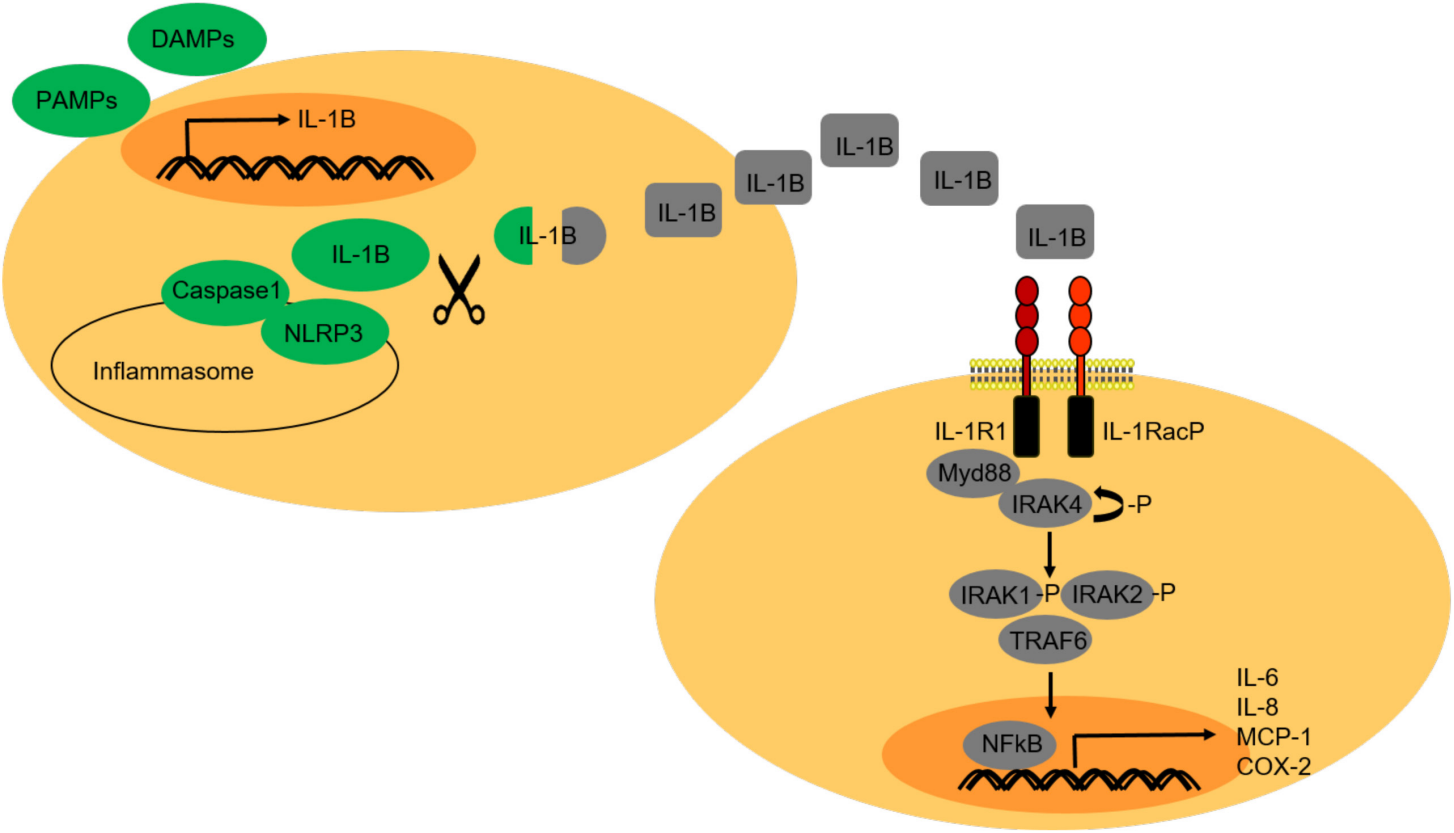
IL-1B signalling. IL-1B is produced as pro-peptide and activated by cleavage upon cell stimulation by PAMPs and DAMPs, as response mechanism to invading pathogens and cellular stress caused by sterile danger signals. IL-1B is functionally activated upon cleavage by caspase-1, which in turn, is activated by the NLRP3 inflammasome complex. After cleavage and activation, IL-1B is released by the cell, according to a poorly understood mechanism that comprehends both active and passive processes, including pyroptosis. Once released, IL-1B activates IL-1R1 receptor. Downstream signalling activation through Myd88, IRAKs and TRAF6 leads to NFKB-mediated transcription of pro-inflammatory cytokines and initiation of inflammation.

The IL-1 type cytokine family comprises seven molecules with agonist functions, including IL-1B and IL-1A, three receptor antagonists and one anti-inflammatory cytokine. Evolution studies have shown that, starting from fish, vertebrates have a single IL-1 gene (Ogryzko* et al*. 2014). IL-1 cytokines bind to IL-1R receptors. There are eleven IL-1R receptors. IL-1B and IL-1A are encoded by distinct genes and bind both to IL-1R1, found on nearly every cell and IL-1R2 (Garlanda* et al*. 2013). IL-1A is also known as alarmin, and it can be found in homeostatic conditions. IL-1A precursor is constitutively present in several epithelial layers, and it is already active when released upon cell necrosis. IL-1A mediates the early phases of sterile inflammation by rapidly inducing a cascade of several cytokines and chemokines. Different from IL-1A, IL-1B is not present in homeostatic conditions. IL-1B plays its main role as ‘gatekeeper’ of inflammation. IL-1B is mainly produced by blood monocytes, tissue macrophages, skin dendritic cells and brain microglia (Garlanda *et al*. 2013). IL-1B synthesis occurs upon stimulation by pattern-associated molecular patterns (PAMPs), whereas its activation is linked to secondary stimuli, such as damage-associated molecular patterns (DAMPs), in the presence of invading pathogens and upon sterile danger signals (Brough* et al*. 2011, Baroja-Mazo* et al*. 2014). IL-1B is processed by the inflammasome, a multi-protein complex activated via non-obese diabetic (NOD)-like receptor (NLR) family, pyrin domain-containing proteins (NLRPs) and required for the activation of caspase 1, needed for IL-1B cleavage and activation (Miller* et al*. 1993, Li* et al*. 1995) (Fig. 2). Caspase-1-mediated pyroptosis is a lytic form of cell death and an innate immune effector mechanism. During inflammation, after caspase-1-mediated cleavage and activation, IL-1B is released in a still poorly understood ‘unconventional’ secretory manner (Vince & Silke 2016). A simple hypothesis suggests that activated IL-1B is passively released after caspase 1-induced pyroptosis, underlining the link between cell death and inflammation. In addition, the role of gasdermins D (GSDMD) proteins in inducing pyroptosis, upon inflammatory caspase cleavage, has been recently described and suggested that their pore-forming function during pyroptosis is needed for IL-1B secretion after maturation (Ding* et al*. 2016). As GSDMs play an important role in the induction of pyroptosis, this type of cell death has been renamed as GSDM-mediated programmed necrosis, characterised by osmolytic potential disruption and consequent cell lysis (Shi* et al*. 2017). The formation of GSDM-mediated pores allows IL-1B secretion even when a cell is still intact and functional, restricting the passage of larger molecules and organelles. Importantly, after pore formation, cells can shut down the inflammasome, allowing IL-1B release while preventing pyroptosis, therefore explaining how IL-1B secretion can occur even without cell death (Kovacs & Miao 2017). Pyroptosis also occurs in non-immune cells, where it is characterised by the activation of other caspases (11/4/5), which do not cleave IL-1B (Shi *et al*. 2017). Alongside caspase-1-mediated pyroptosis, active release of IL-1B has also been described, via secretory lysosomes, multivesicular bodies and direct micro-vesicular shedding (Vince & Silke 2016). Once cleaved and released, IL-1B binds to its correspondent receptor, IL-1R1, which can also be recognised by IL-1A and the antagonist (IL-1RA). However, different from IL-1A and IL-1B, IL-1RA does not trigger downstream signalling activation upon binding to IL-1R1 (Weber* et al*. 2010). Together with IL-1RA, IL-1R2 (decoy receptor) and the ‘shed’ domains of each extracellular IL-1 receptor chains function as negative regulators of IL-1 signalling. IL-1R1 and IL-1R2 share high similarity and their extracellular domains are members of the immunoglobulin superfamily, each comprising three IgG-like domains. The extracellular domains of IL-1R1 and IL-1R2 can also be found as soluble forms. IL-1R has a single transmembrane domain and a cytoplasmic domain and is the main receptor that transduces downstream signalling (Dinarello 1997). IL-1 signalling activation occurs upon binding to the IL-1R1 receptor and consequent conformational change of the extracellular domain. Further recruitment of IL-1RacP (IL-1R-associated protein) and activation of cytosolic Toll- and IL-1R-like domains results in the recruitment of Myd88 (myeloid differentiation primary response gene-88) and IRAK4 (interleukin-1 receptor associated kinase-4). IRAK4 undergoes autophosphorylation and causes the phosphorylation of IRAK1 and IRAK2, with following recruitment of TRAF6 (TNF receptor-associated factor). IRAK1/2 and TRAF6 dissociate from the receptor complex and activate nuclear factor kappa-light-chain-enhancer of activated B cells (NFkB). Once in the nucleus NFkB acts as a transcription factor for inflammatory genes, such as IL-6, IL-8, MCP-1 and cyclooxygenase-2 (COX-2) (Weber *et al*. 2010) (Fig. 2).

### IL-1 signalling in primary tumours and its role in initiating metastasis

Human cancerous breast tissue expresses IL-1B. Recent evidence from our group has shown that breast cancer cells with increased ability to metastasise in bone (MDA-IV) display higher IL-1B expression, compared to its correspondent parental line, suggesting that IL-1B expression correlates with enhanced metastatic potential (Nutter *et al*. 2014).

A positive correlation between IL-1B expression, OPG and CCL2 has been found in human breast cancer genome-wide mRNA expression array. OPG belongs to the TNF receptor superfamily, and it functions as a decoy receptor during bone resorption and affects TRAIL-mediated apoptosis. Inhibition of OPG in breast cancer cell lines showed reduced invasion and metastasis. In particular, IL-1B upregulates OPG via p38 and p42/22 MAPK signalling pathway and induces OPG secretion, which also associates with macrophage infiltration in primary breast tumours. Co-culture of IL-1B-secreting macrophages and breast cancer cells results in enhanced OPG secreted by tumour cells (Chung* et al*. 2017). Taken together, these data suggest a role of IL-1B and OPG in mediating breast cancer metastasis and inflammation. Data shown above suggest that OPG has the potential to act as both pro- and anti-tumour molecule dependent on whether the tumour is at its primary or metastatic site and if OPG is produced by tumour cells or by the cells in the microenvironment. Whether anti-tumour effects are specific to the bone microenvironment remain to be elucidated. OPG, mainly expressed by osteoblasts, is a decoy receptor and is a natural inhibitor of osteoclastogenesis (through inhibition of RANKL/RANK signalling) (Boyce & Xing 2008, Le Pape *et al*. 2016). This potentially explains its anti-tumour effect in bone. In addition to playing a role in tumour growth, IL-1B has been reported to control tumour invasion. *In vitro* evidence from MCF7 breast cancer cells suggests that this may, in part, be due to, IL-1B-regulating breast cancer cell invasion by stimulating production of MMP-9 via focal adhesion kinase 1 FAK and the proto-oncogene tyrosine-protein kinase Src (Mon* et al*. 2017).

IL-1B and IL-1A may both play roles in tumour formation and progression to metastasis. IL-1B has been linked with poor prognosis in breast cancer (Nutter *et al*. 2014), whereas tumour-induced IL-1A has been linked to CCL22 production by tumour-infiltrating immune cells. CCL22 is known to induce the recruitment of T regulatory (Treg) cells. Inhibition of IL-1R1 signalling using Anakinra has been found to impair Treg migration (Wiedemann* et al*. 2016). In the same study, the authors also showed that as a result of silencing IL-1A in pancreatic tumour cells (PaTu), CCL22 production was inhibited in PBMC, whereas inhibition of IL-1A or IL-1B in mammary 4T1 cancer cell line resulted in impaired CCL22 production in splenocytes. In addition to promoting tumour progression, by controlling Treg recruitment via CCL22 signalling, however, IL-1A mediated IL-1R1 signalling has also been reported to have anti-tumour functions. In particular, using a spontaneous murine mammary tumour model (MMTV-PyMT), Dagenais and colleagues have shown that IL-1R1 signalling suppresses mammary tumour cell proliferation early in tumourigenesis and reduces breast cancer outgrowth and metastasis in the lungs. High primary tumour burden was found in both IL-1R1 −/− and IL-1A −/− mice, and this phenotype was independent of caspase-1/-11 (Dagenais* et al*. 2017). Hence, IL-R1 signalling could have both pro- and anti-tumour functions and it is possible that IL-1A and IL-1B may exert opposite functions in breast cancer progression.

The murine spontaneous mammary tumour model MMTV-PyMT has been employed to investigate the role of IL-1 and the inflammasome in primary mammary tumour growth and pulmonary metastasis, alongside allograft and xenograft models of breast cancer. For this purpose, mice lacking functional caspase-1 and NLRP3 in combination with pharmacological treatment to inhibit IL-1R were employed. Inhibition of the inflammasome and IL-1 signalling resulted in reduced primary tumour growth, lung metastasis and myeloid cell infiltration (Guo* et al*. 2016).

**Figure 3:**
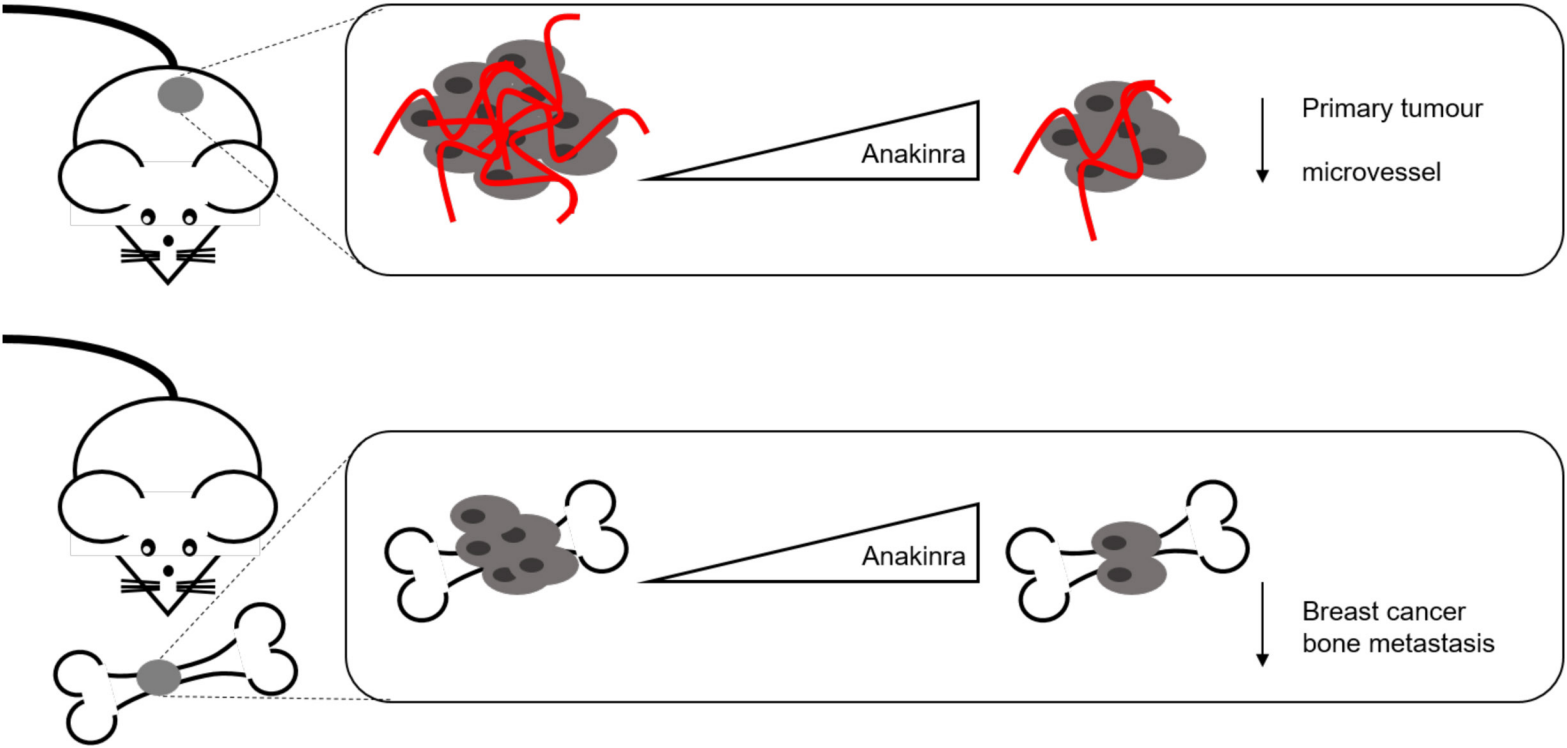
IL-1 signalling in breast primary tumour and bone metastasis. IL-1 signalling controls breast cancer growth at both primary and bone metastatic sites. Osteotrophic breast cancer cells MDA-231-IV or ER+ breast tumour cells MCF-7 are implanted subcutaneously in BALB/c-nude mice to mimic primary tumour growth. IL-1 signalling inhibition using the receptor antagonist Anakinra results in reduced tumour growth as well as diminished tumour microvessel density. Upon i.v. injection, MDA-231-IV cells home to bone. Anakinra treatment affects metastatic breast cancer tumour growth, without altering the number of cells homing to this site, suggesting the involvement of IL-1 signalling in breast cancer dormancy in bone.

The data above fit with our recent findings in which we showed that IL-1 associates with primary tumour development of human mammary tumours *in vivo*. Pharmacological inhibition of IL-1R with Anakinra before subcutaneous injection of either triple-negative MDA-231-IV or ER+ve MCF-7 cells significantly reduced tumour formation, whereas treatment of established tumours with Anakinra significantly reduced tumour cell proliferation (Fig. 3). These data suggest that inhibition of IL-1R signalling impairs breast cancer progression from both ER-ve and ER+ve tumours (Holen* et al*. 2016*a*).

IL-1B has been shown to have profound effects on tumour growth and metastasis in tumour types other than breast cancer, including melanoma. A reduction of local tumours or lung metastasis was found in mice lacking functional IL-1R signalling (IL-1B KO), together with diminished tumour angiogenesis. Similar results on angiogenesis were also found upon IL-1R pharmacological inhibition. IL-1A KO mice also displayed a reduction in melanoma growth and angiogenesis compared to WT animals, but the effect was less evident when compared to IL-1B KO mice, suggesting a major role of IL-1B. The findings could be extended to mammary and prostate cancer models (Voronov* et al*. 2003).

### IL-1B signalling in tumour-induced angiogenesis

Tumour angiogenesis is a hallmark of cancer and IL-1 is a strong endothelial cell (EC) activator. IL-1 enhances pro-inflammatory responses, angiogenesis, growth rate, migration, morphological changes, contact inhibition and permeability of ECs. IL-1B upregulates VEGF and its correspondent receptor on ECs and via IL-1R1 causes EC migration and tube formation by activating p38-MAPK and MAPK-activated protein kinase 2. Moreover, IL-1 has been described to synergise with VEGF and overlapping gene signatures have been identified in ECs, particularly for genes involved in cell growth and inflammation. In a matrigel model, abrogation of rVEGF or rIL-1B resulted in an abolished angiogenic response. Administration of rVEGF to mice with impaired IL-1B signalling resulted in reduced neo-angiogenic response in matrigel plugs (Carmi* et al*. 2013). There is considerable interest in using anti-angiogenic therapy; however, the results from clinical trials have been disappointing due to tumours rapidly developing resistance mechanisms to these drugs. The tumour microenvironment plays a large role in resistance to anti-angiogenic drugs with immune cells being a predominant source of angiogenic factors. Consequently, there are now a multitude of studies looking at the effect of combinatorial treatments to block angiogenesis as well as to modify immune cell response to improve the efficacy of anti-angiogenic therapy. Inhibition of IL-1B impairs the recruitment of immune cells at cancerous sites, limiting the production of pro-angiogenic molecules. IL-1Ra has been found to inhibit the angiogenic response, concomitantly inhibiting tumour progression. We recently showed that IL-1R1 antagonist Anakinra reduces the number of microvessels in sub-cutaneous breast tumours and bone metastases caused by the osteotrophic triple-negative breast cancer cell line MDA-MB-231-IV, after both a preventative and treatment strategy (Fig. 3) (Holen *et al*. 2016*a*). Therefore, IL-1 signalling inhibition in combination with anti-angiogenic therapies could represent the way forward to limit tumour progression (Voronov *et al*. 2003).

### IL-1B signalling in breast cancer and inflammation

IL-1B signalling and inflammation are strongly connected and inflammation plays a major role during cancer progression. In particular, the fine balance between IL-1B and IL-1Ra activity is important to control inflammation: IL-1B and IL1R1a overexpression results in an enhanced and reduced inflammatory state, respectively.

In bone inflammatory diseases like rheumatoid arthritis, enhanced inflammation and osteoclast differentiation and activation have been reported. In this condition, enhanced infiltration of macrophages, which are a primary source of pro-inflammatory cytokines, causes bone damage (Okano* et al*. 1996, Funk* et al*. 1998). In addition, IL-1B produced by macrophages and T cells increases osteoclastogenesis, by regulating osteoclast progenitors (Strand & Kavanaugh 2004). Because IL-1B regulates bone homeostasis and contributes to enhanced bone resorption upon inflammation, it is possible to speculate that in breast cancer metastasis, IL-1B alters bone turnover in favour of the vicious cycle (Fig. 1). The mechanism by which IL-1B produced by different cell types promotes metastasis is still under investigation.

Immune cells also play a pivotal role in cancer and inflammation. Mice lacking IL-1R display delayed myeloid-derived suppressor cell (MDSC) infiltration and reduced primary breast tumour development, a phenotype that is restored by IL6. Conversely, mice with a non-functional IL-1Ra display enhanced inflammation, characterised by a more rapid accumulation of MDSCs in breast tumour. This finding suggests that inflammation supports tumour progression by enhancing immune-suppressive mechanisms (Bunt* et al*. 2007). *In vitro* studies have recently shown that primary breast cancer cells co-cultured with monocytes display increased IL-1B, IL8 and MMPs (Espinoza-Sanchez* et al*. 2017), suggesting that inflammation and therefore the recruitment of immune cells support the development of breast cancer during the early stages. In addition, pro-inflammatory effects of IL-1B also promote metastasis: In a spontaneous *in vivo* model of breast cancer, tumour cells induce systemic inflammation by producing IL-1B, which stimulates IL-17 production by γδ T cells. Subsequently, IL-17 drives the production of G-CSF that causes neutrophil expansion and polarization towards a CD8+ T cell-suppressive phenotype via iNOS, enhancing immune suppression and distant metastases (lung and lymph nodes) (Coffelt* et al*. 2015). IL-1B is also involved in the mobilisation of MDSCs further promoting both primary and metastatic breast tumours (Karki* et al*. 2017).

Finally, NLRP3-IL-1B-IL1R signalling has been shown to control NK and T cells, by inhibiting their tumouricidal potential and inhibiting the anti-tumour effect of chemotherapeutic agents by inducing the secretion of IL17 by CD4+ T cells (Karki *et al*. 2017). The IL-1B-driven detrimental role of the inflammasome in cancer opposes to its protective role, mainly driven by IL-18, as reviewed in (Karki *et al*. 2017).

### IL-1B in breast cancer bone metastasis

Expression of IL-1B by primary breast cancer cells strongly correlates with disease re-occurrence and future relapse in bone: In tissue arrays containing 150 primary tumour biopsies taken from patients newly diagnosed with stage II/III breast cancer enrolled onto the AZURE trial, 49% of patients with detectable IL-1B in their tumours experienced relapse compared with 7% of patients with IL-1B-negative tumours and 37% of patients with detectable IL-1B in their primary tumours developed bone metastasis compared with 5% of patients with IL-1B-negative tumours over an 84-month time period (Coleman* et al*. 2011). In addition, breast cancer cell lines that specifically home to and colonise mouse bone (MDA-IV) have been shown to express high levels of IL-1B (Nutter *et al*. 2014). We have recently shown that inhibiting IL-1R signalling, using the receptor antagonist Anakinra, results in decreased breast cancer bone metastases (Fig. 3). Administration of Anakinra before injection of tumour cells did not prevent numbers of tumour cells that arrived in the bone environment, but held these tumour cells in a dormant state, significantly inhibiting development of both micro-metastases and overt metastases. When Anakinra was given 1 week after tumour cell inoculation these existing bone metastases ceased to grow, and this appeared to be the result of reduced proliferation and reduced angiogenesis, whereas apoptotic cell death remained unaltered. Because of the tight coupling between tumour cells and bone cells that occurs in bone metastases, the effects of IL-1B on the bone environment may also play important roles in this process. In tumour-naïve mice, a single dose of Anakinra reduces osteoclast and osteoblast activity in addition to causing a reduction in IL-1B and TNFa levels indicating that in addition to anti-tumour effects of Anakinra, this drug may also target bone cells thus reducing resorption and availability of bone-derived growth factors that can further stimulate tumour growth. These Anakinra-induced alterations to the bone environment were maintained following continuous administration of Anakinra for >21 days, and this treatment did not cause significant effect on trabecular bone volume. Taken together, these data suggest that Anakinra could be re-purposed as a treatment of bone metastatic breast cancer (Holen *et al*. 2016*a*).

As evidenced above, blocking IL-1R activity with Anakinra holds disseminated tumour cells in a dormant state in the bone environment. Recent *in vitro* data indicate that IL-1B and not IL-1A is the relevant cytokine linked to dormancy of breast cancer cells in this environment. Co-culture of a human breast cancer cell line, with suppressed ability to form metastases, with osteoblasts resulted in faster growth of breast cancer cells attached to the matrix made by the osteoblasts when stimulated with IL-1B and TNFa. Interestingly, the IL-1B and TNFa-stimulated growth was suppressed when COX2 activity or PGE2 receptor were blocked, in line with data showing that IL-1B and TNFa activate the arachidonic acid pathway (Mark* et al*. 2001, Ono 2008, Sosnoski* et al*. 2015).

MSCs play a pivotal role in bone metastases as their niche overlaps with the cancer stem cell niche in this environment. How breast cancer cells, chemokines and cytokines, including IL-1B affect these cells may be a fundamental factor in determining whether tumour cells in bone are able to progress to overt metastases. Exposing MSCs to conditioned media derived from metastatic breast cancer cells resulted in increased levels of chemokines, such as CXCL1, 3, 5, 6, 8, CCL2 and CCL7. Importantly, chemokine expression was inhibited in MSCs exposed to conditioned media derived from metastatic breast cancer cells treated with NFKB inhibitor. Interestingly, amongst NFKB downstream targets, IL-1B has been identified as a major regulator of chemokine production in MSCs. IL-1B was present in the supernatant of metastatic breast cancer cells. When treated with IL-1B, MSCs were found to upregulate the same cytokines that were observed following exposure to cancer cell conditioned media. Inhibition of IL-1B in metastatic breast cancer cells resulted in no chemokine production by MSCs, whereas overexpression of IL-1B in non-metastatic breast cancer cells increased chemokine levels in MSCs. Therefore, IL-1B is a major candidate in driving the production of chemokines in the tumour microenvironment, which, in turn, sustain tumour development (Escobar* et al*. 2015).

## Murine models of breast cancer bone metastasis for investigating IL-1B signalling

The majority of mouse models that are used to study bone metastasis involve injecting MDA-MB-231 cells (breast) or PC3 (prostate) and their bone-homing derivatives directly into the left cardiac ventricle from where the cells become disseminated in the skeleton (Ottewell* et al*. 2010, 2014*a*,*b*, Nutter *et al*. 2014). Although this method does not mimic the early stages of the metastatic process consisting of growth at the primary site and dissemination into the circulation, human breast cancer cell lines do not spontaneously metastasise from the fat pad to the mouse skeleton without oestradiol supplementation. Although oestrogen receptor-positive breast cancer cell lines, MCF7 and T47D reliably metastasise from mouse mammary fat pads to mouse long bones in ~90% of animals when mice are supplemented with oestradiol, it is hypothesised that metastasis to bone is driven by the bone anabolic effects of oestradiol (Holen* et al*. 2016*b*), as IL-1B also affects bone turnover data obtained using these model systems would need to be interpreted with this in mind (Holen *et al*. 2016*a*). For more basic experiments designed to investigate tumour growth in bone and tumour cell–bone cell interactions, it is possible to inject most cancer cell lines directly into the tibiae or femur. It must be noted, however, that this method does not model tumour cell dissemination into the bone metastatic niche, tumour cells grow randomly within the marrow cavity from direct injection into bone and bone healing occurs in conjunction with tumour induced lesion formation, complicating data analysis (Wright* et al*. 2016)

The growth of human cancer cells in mice requires the use of immunocompromised mice negating the role of the immune system. This may be especially important when studying the effects of a pro-inflammatory cytokine such as IL-1B. Intra-cardiac or intra-mammary injection of mouse mammary 4T1 cells results in metastasis to lung, liver, brain and bone and bone homed derivatives including 4T1.1 have increased bone-homing capacities. Many researchers report increased metastasis following removal of the mammary tumour (Francia* et al*. 2011). The use of syngeneic models is an excellent tool to study the interaction between tumour cells and the microenvironment primarily because these animals have a fully competent immune system.

The use of breast cancer cell lines to model cancer development and progression has been heavily criticised due to these cells having been cultured in the laboratory for numbers of years leading to culture adaptation and loss of heterogeneity. This has led to the development of mouse models of metastasis utilising patient-derived tumours. Surgical implantation of patient-derived xenografts (PDXs) orthotopically into mice has been successfully used to study bone metastasis from prostate, breast and lung cancer, as well as other metastatic sites for different cancer types (i.e. lymph node and liver metastasis for colon cancer and metastatic pancreatic, stomach, ovarian, bladder and kidney cancer) (Hoffman 1999). Tumour cells behave differently according to the microenvironment in which they are seeded. Orthotopic models offer significant advantages over subcutaneous models as they more accurately model interactions between tumour cells and organ-specific microenvironments, allowing to study the behaviour of human tumours in the tissue from which they originate. Environmental signals prime tumours in the primary site to metastasise to specific organs, therefore, in order to accurately study organ-specific tropism of metastasis, orthotopic implantation of tumour cells may be necessary. However, bone metastasis from these models is rare and growth of patient-derived material requires oestradiol supplementation, and the bone anabolic effects of oestradiol need to be taken into account when interpreting data from these models.

Other PDX models have been developed to address different questions related to the biology of cancer. PDX allows to study intra- and inter-tumour heterogeneity and how this affects response to therapy. PDX models can be used to study primary or acquired resistance to drugs by engrafting patient-derived material that has been isolated from patients before or after therapy, respectively. Clinical trial-associated xenografts (CTAXs) are a form of PDX model that has the potential to be used in personalised medicine, as biopsies taken from patients during clinical trials, at different stages of treatment and disease progression. Generally, PDX models use immunocompromised animals to allow engraftment of human tissues. Originally, patient-derived xenografts to study metastatic breast cancer were developed in nude mice (Fu* et al*. 1993), whereas today there is a conscious move to using murine models of NOD SCID gamma mice, which have been engrafted with human immune cells. In this model, total peripheral blood or bone marrow from healthy donors or patients is injected into the tail vein of young animals in order to consider the contribution of the immune system in tumour progression.

There are a number of mouse models that can be used to study different stages of the bone metastatic process from different tumour types. Each model has its pros and cons and data obtained from these models should be interpreted with specific limitations in mind. There is an ongoing effort to make more clinically relevant mouse models of bone metastasis and a complete review on how to set up and utilise these models can be found in Wright* et al*. (2016).

## Pharmacological inhibition of IL-1 signalling

IL-1B is an inflammatory molecule and targeted therapies have been developed to treat chronic inflammation and auto-inflammatory diseases. IL-1 signalling inhibition was first achieved using Anakinra, a recombinant form of the naturally occurring IL-1 receptor antagonist. Originally introduced in 2001 to treat rheumatoid arthritis (Dinarello* et al*. 2012), Anakinra has been recently proposed as a potential treatment for pericarditis and cardiovascular complications occurring during diabetes mellitus (Peiro* et al*. 2017). It is currently clinically indicated for the treatment of inherited auto-inflammatory diseases associated with non-functional NLRP3 and ILRN cryopirin-associated periodic syndromes (CAPS) (Jesus & Goldbach-Mansky 2014). Alongside Anakinra, Rilonacept (soluble decoy receptor) and Ilaris (or Canakinumab, a neutralizing anti-IL-1B IgG1 antibody) are the drugs currently used in the clinical practice to target the IL-1 signalling pathway (Peiro *et al*. 2017). As shown in Table 1, Anakinra is currently in clinical trials for metastatic breast cancer (Nbib1802970), metastatic colorectal cancer (Nbib2090101), pancreatic adenocarcinoma (Nbib2550327), multiple myeloma and plasma cell neoplasm (Nbib635154). It is used alone or in combination with chemotherapeutic agents (Gemcitabine, Nab-Paclitaxel, Cisplatin) (Nbib2550327), anti-resorptive agents (Denosumab, anti-RANKL monoclonal antibody) (Nbib1624766), mTOR inhibitor (Everolimus) (Nbib1624766) and Lenalidomide and Dexamethasone (Nbib2492750) (early stages multiple myeloma). A clinical trial for colorectal cancer, triple-negative breast cancer, NSCLC and adenocarcinoma in Phase 1 is currently recruiting participants to test the checkpoint inhibitor PDR001 in combination with other immunomodulatory agents, including Ilaris (Nbib2900664). The results of these clinical trials will be awaited with interests and they will provide valuable information on the use of Anakinra and Ilaris used alone or in combination with other therapeutic regimes to treat cancer patients.

**Table 1:** Clinical trials involving the use of Anakinra and Ilaris for the treatment of different cancer types.

Treatment	Cancer type	Clinical trial identifier	Clinical trial phase
Anakinra and standard of care	Metastatic breast cancer	Nbib1802970	Phase I
Anakinra and Bevacizumab	Metastatic colorectal cancer	Nbib2090101	Phase II
Gemcitabine, Nab-Paclitaxel, Cisplatin and Anakinra	Pancreatic adenocarcinoma	Nbib2550327	Early Phase I
Anakinra alone or with Dexamethasone	Multiple myeloma	Nbib635154	Phase II
	Plasma cell neoplasm		
Anakinra or Denosumab and Everolimus	Advanced cancers	Nbib1624766	Phase I
Lenalidomide and Dexamethasone with or without Anakinra	Indolent plasma cell myeloma	Nbib2492750	Phase I, followed by Phase II
	Plasma cell myeloma		
	Smoldering plasma cell myeloma		
PDR001 in combination with CJM112, EGF816, Ilaris (Canakinumab) or Mekinist (Trametinib)	Colorectal cancer, triple-negative breast cancer, NSCLC, adenocarcinoma	Nbib2900664	Phase I

## Conclusions and future directions

Bone is a preferential metastatic site for breast cancer. IL-1B has been identified as biomarker that can be used to predict which breast cancer patients are likely to experience relapse in bone. *In vitro* studies have suggested so far that IL-1B correlates with increased aggressiveness of breast cancer cell lines. Although *in vivo* studies are in their infancy, those that have been carried out confirm these findings. In particular, we have shown that IL-1 signalling is linked to breast cancer metastasis specifically to bone. The inhibition of IL-1R1 and reduced bone metastasis in our mouse model shows the role of this signalling axis in breast cancer metastasis to bone, but further studies are needed to elucidate whether IL-1B and/or IL-1A are primarily involved in this process.

The combination of anti-angiogenic therapies and immune therapies represent a new strategy to treat tumours that develop resistance to standard of care treatments and/or anti-angiogenic therapy alone. Intra-tumoural myeloid cells have been found to regulate responsiveness and resistance to anti-angiogenic therapies (Rivera* et al*. 2015). IL-1B is the gatekeeper of inflammation. It is produced mainly by immune cells, challenged by invading pathogens and danger signals and has been found to exert angiogenic functions. Therefore, inhibiting IL-1B signalling is a potential new therapeutic strategy to simultaneously block angiogenesis and dampen inflammation, impairing breast cancer bone metastasis. The availability of safe and approved therapeutic strategies targeting IL-1 signalling such as Anakinra and Ilaris could facilitate the treatment of patients affected by breast cancer-driven bone disease.

## Declaration of interest

The authors declare that there is no conflict of interest that could be perceived as prejudicing the impartiality of this review.

## Funding

Research into the role of IL-1B in breast cancer bone metastasis is funded by research grants from the MRC (Grant: MR/P000096/1) and Weston Park Cancer Charity (Grant: CA142) both awarded to P O.
